# Prevalence, spatial and temporal distribution of tungiasis in the Kilifi Health and Demographic Surveillance System (KHDSS) in Kenya

**DOI:** 10.1136/bmjgh-2025-020057

**Published:** 2026-03-04

**Authors:** Nelson Ouma, Samuel K Muchiri, Christopher Nyundo, David Walumbe, Amek Nyaguara, Marta Maia, Ifedayo Adetifa, Benedict Orindi, Phillip Bejon, Ulrike Fillinger, Lynne Elson

**Affiliations:** 1 KEMRI-Wellcome Trust Research Programme, Kilifi, Kenya; 2 Population and Health Impact Surveillance Group, KEMRI-Wellcome Trust Research Programme, Nairobi, Kenya; 3 Nuffield Department of Medicine, University of Oxford, Oxford, UK; 4 London School of Hygiene and Tropical Medicine, London, UK; 5 International Centre of Insect Physiology and Ecology, Nairobi, Kenya

**Keywords:** Tungiasis, Kenya, Epidemiology, Global Health, Geographic information systems

## Abstract

**Introduction:**

Tungiasis is a highly neglected tropical disease of the skin caused by an embedded female sand flea affecting the most resource-poor communities in sub-Saharan Africa, the Caribbean and South America. The global disease burden is unknown and systematic, fine-resolution spatial data on prevalence and environmental and ecological risk factors are rare.

**Methods:**

We leveraged the Kilifi Health and Demographic Surveillance System of 90 257 households and asked whether they had a case of tungiasis in the household at interview during three survey rounds of routine surveys, undertaken between May 2021 and May 2022. Precise geospatial data to locate households were matched to macrolevel environmental, ecological and soil covariates, and multilevel logistic regression models were used to test for associations.

**Results:**

A total of 1376 (1.5%) households reported a case in at least one survey during the year, while only 25 households did for all three surveys. The prevalence decreased over the three rounds from 1.1%, through 0.5–0.2%. The odds of having a tungiasis case in a household were higher in houses with earthen floors and walls, and in rural locations. The odds increased with increases in the number of children in a household and with population density (within 1 km radius), rainfall, Enhanced Vegetation Index, land surface temperature, aridity, altitude and organic carbon in the soil. However, the odds of having a tungiasis case in a household decreased with increasing aluminium content in the soil. These factors accounted for 23.9% of the variability in tungiasis distribution by household.

**Conclusion:**

Tungiasis distribution was heterogenous and changed over time. Macro level environmental factors predicted the niche maps for tungiasis and could have applications in guiding local surveys and interventions.

**WHAT IS ALREADY KNOWN ON THIS TOPIC**

- The disease is known to occur in multiple countries in Latin America and sub-Saharan Africa but has a heterogeneous distribution with the prevalence in targeted communities ranging from 7% to 63% and main risk factors being linked to poverty.

**WHAT THIS STUDY ADDS**

- We demonstrate a heterogenous distribution of tungiasis with affected households clustered in the southern part of the geographical area covered, a change in prevalence over the year and changes in which households were affected.
- Having a tungiasis case in the household was associated with population density, rainfall, land surface temperature, vegetation cover, altitude, soil aluminium and organic carbon content, along with house floor and accounted for 23.9% of the variability in the disease distribution.

**HOW THIS STUDY MIGHT AFFECT RESEARCH, PRACTICE OR POLICY**

- Environmental factors can be used to predict the possible distribution of tungiasis but because control activities, socio-economic status and behavioural factors also play a role, targeting public health interventions will need targeted localised surveys.

## Introduction

 Sand flea disease (tungiasis) is a highly neglected parasitic skin disease caused by the sand flea, *Tunga penetrans*, which is often referred to as ‘jiggers’ in Kenya. This parasite inflicts pain and suffering on millions of impoverished people in South America, the Caribbean and sub-Saharan Africa.^1^ The adult female flea burrows into the skin, mostly on the feet, where it grows 2000-fold in size over 7 days as the eggs develop.^2^

Research on tungiasis is very limited and tungiasis has only recently become formally recognised by the WHO as a neglected tropical disease (NTD).^3 4^ The disease affects communities living in precarious conditions, trapping them in a vicious cycle of poverty and disease.^1^ Awareness of the public health importance of tungiasis has been growing in East Africa in recent years,^5^ but data on epidemiological characteristics, necessary for the planning and implementation of a control programme, are scarce. In Kenya, the national prevalence among children aged 8–14 years was 1.35% in one study in 2021,^6^ but other studies in Kenya have documented a prevalence range between 19% and 40%.^7^

When the geographical distribution of NTDs is well understood, the targeting of control measures is greatly improved.^8^ Considerable progress on mapping of many NTDs has been made^9 10^ but not for tungiasis. A previous study produced a map of tungiasis risk across Africa at a coarse scale, but was limited by the availability of data and did not include any fine-grained geospatial data of individual homesteads.^11^

The Kenya Medical Research Institute (KEMRI)-Wellcome Trust Research Programme implements a well-established Health and Demographic Surveillance System (KHDSS) in Kilifi County in coastal Kenya^12^ which provided a unique opportunity to incorporate a survey question to determine the prevalence, spatial and temporal distribution, and macrolevel environmental risk factors associated with tungiasis.

## Methods

### Study site and design

**Figure 1:**
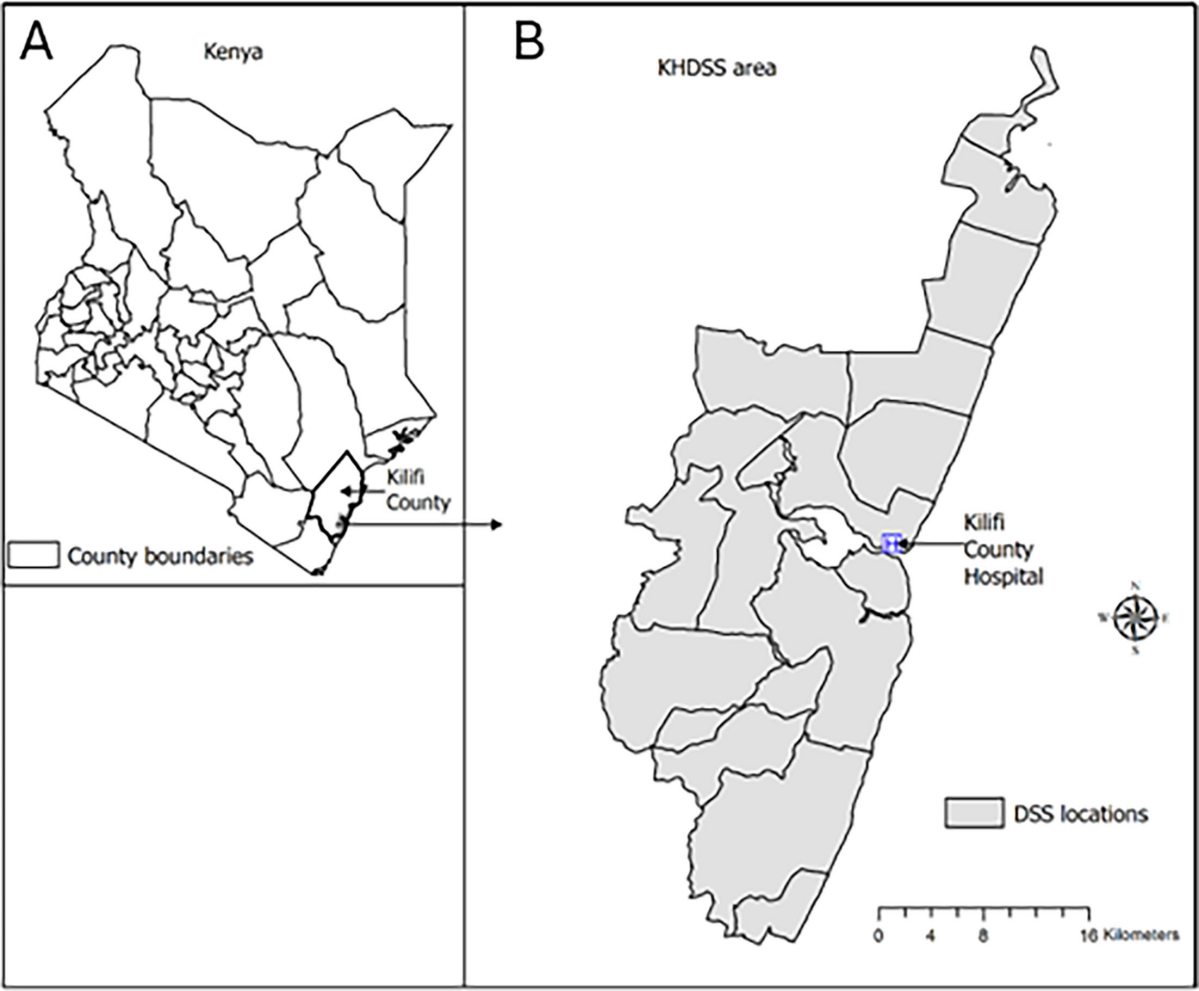
Location of the study site. Position on the coast of Kenya (**A**) and the KHDSS with administrative location boundaries marked (**B**). KHDSS, Kilifi Health and Demographic Surveillance System.

The study was a 1-year longitudinal cohort study of all households in the KHDSS. The KHDSS site is located within Kilifi County in the coastal area of Kenya (figure 1), was established in 2000 and covers an area of 891 km^2^.^12^ The altitude varies from sea level along the eastern border to a maximum of 255 m on the western border. The area experiences two rainy seasons (April to July and October to December) with an average annual rainfall of between 900 and 1000 mm and temperatures from 22°C to 32°C. The population is served by Kilifi County Hospital which is located at the centre of the KHDSS area.

The KHDSS was initially established to determine rates of fertility, mortality and migration by routinely monitoring demographic (birth, death, pregnancy, migration) events at household level in a geographically defined area. This is done through 4-monthly surveys every year to every building unit. Intermittent surveys are added to provide information on socio-economic development and health topics such as vaccine coverage, malaria and epilepsy incidence.^12^ All dwellings have been global positioning system (GPS) mapped, and all residents are invited to participate with no exclusion criteria, with less than 1% declining to do so.^12^

Study participants were all occupants of building units within the KHDSS^12^ which at the time had a population of approximately 309 000 people distributed over 90 257 building units within 65 756 homesteads covering 15 administrative locations (figure 1). For the purposes of this manuscript, a building unit is referred to as a household, which is a group of people who mostly sleep in the same building unit and eat together. The majority of the population are of the Giriama tribe who speak their own language as well as Kiswahili, the official language of Kenya, and some speak English. The major economic activity is subsistence farming of maize, cassava, cashew nuts and coconuts, as well as goats and dairy cattle farming.^12^

This study was nested in three of the regular enumeration rounds of the KHDSS (round 1: 4 May to 23 September 2021, round 2: 24 September 2021 to 14 January 2022 and round 3: 15 January to 4 May 2022). One question on tungiasis was added into the routine questionnaire: ‘does anyone in this household currently have tungiasis?’. The question was translated into Kiswahili by a group of field officers and the KHDSS managers, back translated by another manager and verified by the Principal Investigator (PI). The word *‘funza’* was used for tungiasis which is the word used in both Kiswahili and Giriama. In accordance with the KHDSS standard operating procedures, questions were answered by household heads or, where absent, an adult representative from the household. Wherever all were absent, the household was revisited within a few days. If still no adult was found, information was collected from the immediate next neighbour. Information collected from neighbours is verified from the household heads in subsequent enumerations and updated. Piloting of the questionnaire was done as part of the standard procedures within the KHDSS during the first 2 days of data collection. All households in the KHDSS were eligible to participate, but answering the tungiasis question was voluntary. Verbal consent was sought from respondents before information was collected. No clinical examinations were conducted to confirm a tungiasis diagnosis.

### Explanatory variables

#### Household information and tungiasis infection

Data collection was conducted electronically using handheld electronic devices, which had checks and balances embedded in the application to ensure data quality. The devices were password protected to ensure data confidentiality and security. The geographical coordinates (longitude, latitude and altitude) for each household were recorded with a GPS receiver (eTrex 10).

Housing characteristics were collected as proxy indicators of poverty and included roofing, floor and wall material. The construction materials were each recategorised into either improved or unimproved based on the categorisation used by the KHDSS.^12^ Details of the categorisation are included in online supplemental table S1. The population density was calculated for a 1 km radius of each household. The urban/rural classification was that digitised by Macharia *et al*.^13^ They used the urban areas classified by the Kenya National Bureau of Statistics which defines urban areas as an area with high density of people and man-made features such as buildings, and a population of at least 2000 people.^14^

#### Environmental and ecological factors

For geospatial analysis, several environmental and ecological variables were included that were considered to be relevant to the survival and development of the flea off-host stages as guided by available literature.^15^ The environmental variables included the long-term averages for the aridity index obtained from the Consultative Group on International Agricultural Research-Consortium for Spatial Information and altitude data from the Shuttle Radar Topographic Mission digital altitude model. Temporal data were obtained for: Land Use Land Cover (LULC) from the European Space Agency,^16^ the Moderate Resolution Imaging Spectroradiometer for Enhanced Vegetation Index (EVI) and day and night land surface temperature (LST) and night-time lights (NTLs). Average monthly rainfall estimates were obtained from the Climate Hazards Group InfraRed Precipitation with Stations.^17^ LST and rainfall data were collected for each month and combined for each survey round. All the raster values were extracted using ArcMap V.10.8.2 (ESRI, Redlands, California, USA). Details and maps of the environmental variables are included in online supplemental table S2 and figure S1. Altitude was transformed into a four-level ordinal variable: 0, 1–50, 51–100, 101–255 m above sea level.

#### Soil data

The soils data used for the analysis were downloaded from the International Soil Reference and Information Centre.^18^ The soil properties extracted include soil pH in water at a depth of 0 cm, soil texture at a depth of 0 cm, soil carbon content, extractable aluminium content at a depth of 0–30 cm and extractable iron content at a depth of 0–30 cm. All raster datasets were at a spatial resolution of 250 m. The soils data were extracted to the tungiasis surveillance points using the ‘extract multi-value to point’ tool in ArcMap V.10.8.2. (ESRI). Details and maps of the soil variables are included in online supplemental table S2 and figure S1.

### Statistical analysis

Data analysis was conducted using Stata V.17 (Stata Corporation, College Station, Texas, USA). The primary outcome was the presence or absence of at least one infected member in a household as reported by the respondent. We estimated the prevalence of tungiasis together with their associated 95% Wilson CIs (95% CIs). Univariable multilevel logistic regression analysis was performed with household ID as a random effect. Variables significant at p<0.05 were added into the first multilevel multivariable model (Model 1). The final multilevel multivariable model (Model 2) included only the covariates significant at p<0.05 in Model 1 on likelihood ratio (LR) test. Based on a variance inflation factor (VIF) threshold of five, there was no multicollinearity (data not presented). We present final model results as adjusted OR with corresponding p values and CIs. Only households enumerated in all three survey rounds were included in the models to be sure of a full dataset for all households. There were no missing data points for any of the explanatory variables. The very large sample size countered any bias that may have arisen. To assess the variability in the outcome variable associated with each covariate in Model 2, Pseudo R^2^ was calculated from the log-likelihoods as each variable was removed one at a time.

Geospatial analysis was performed using ArcGIS V.10.8.2 (ESRI). GPS coordinates for households with tungiasis were extracted and plotted in ArcMap to show the spatial distribution of tungiasis across the Kilifi HDSS area using the administrative location boundaries base map from the Kenya National Bureau of Statistics.

### Patient and public involvement

Residents of the KHDSS were the instigators of this study. Through their community representatives, who meet regularly with KEMRI researchers, they expressed their desire to have research conducted on tungiasis. Since the study design was to leverage the ongoing routine HDSS, there was no involvement from the community for the tungiasis study specifically. The authors designed the single question that was asked at each household and was then discussed with the community representatives. Dissemination of the results was conducted with county officials and the community representatives.

## Results

### Study participants

The number of households enumerated increased from 89 260 in round one to 91 180 in round three (table 1). A total of 81 990 households were enumerated in all three survey rounds and were included in the risk factor analysis. The 9190 households excluded from the analysis either did not exist in earlier rounds (n=5120), the building existed but had no residents in later rounds (n=3979) or they declined to participate (n=91). Of the 81 990 households, 87.5% were in rural areas, 72.9% had an earthen floor, 57.6% had an improved roof and 19.2% had improved walls. On average, a household in the KHDSS was occupied by seven residents (three adults and four children). Nearly half of all households were at sea level, 35.4% between 1 m and 100 m, and 15.6% were at altitudes between 100 m and 255 m above sea level. The distribution of households by all explanatory variables is detailed in the online supplemental table S3.

**Table 1:** Prevalence of tungiasis by KHDSS round

Survey round	Survey months	Number of households surveyed	Positive responses	Prevalence (%)	95% CI
1	4 May−23 September 2021	89 260	986	1.10	1.04 to 1.17
2	24 September 2021−14 January 2022	90 333	427	0.47	0.43 to 0.52
3	15 January−4 May 2022	91 180	217	0.24	0.21 to 0.27

KHDSS, Kilifi Health and Demographic Surveillance System; tungiasis, sand flea disease.

### Prevalence of tungiasis in the KHDSS

The prevalence of households reporting at least one case of tungiasis decreased over time from 1.1% in round 1 (May–September 2021), through 0.5% in round 2 (September 2021–January 2022) and 0.2% in round 3 (January–May 2022) (table 1). Of the 91 180 households visited at least once, 1.8% reported having a case and of the 1630 households who reported having a case of tungiasis, 90.2% were infected in only one round, 8% in two rounds and 1.8% in all three rounds.

### Spatial and temporal distribution of tungiasis in the KHDSS study area

**Figure 2:**
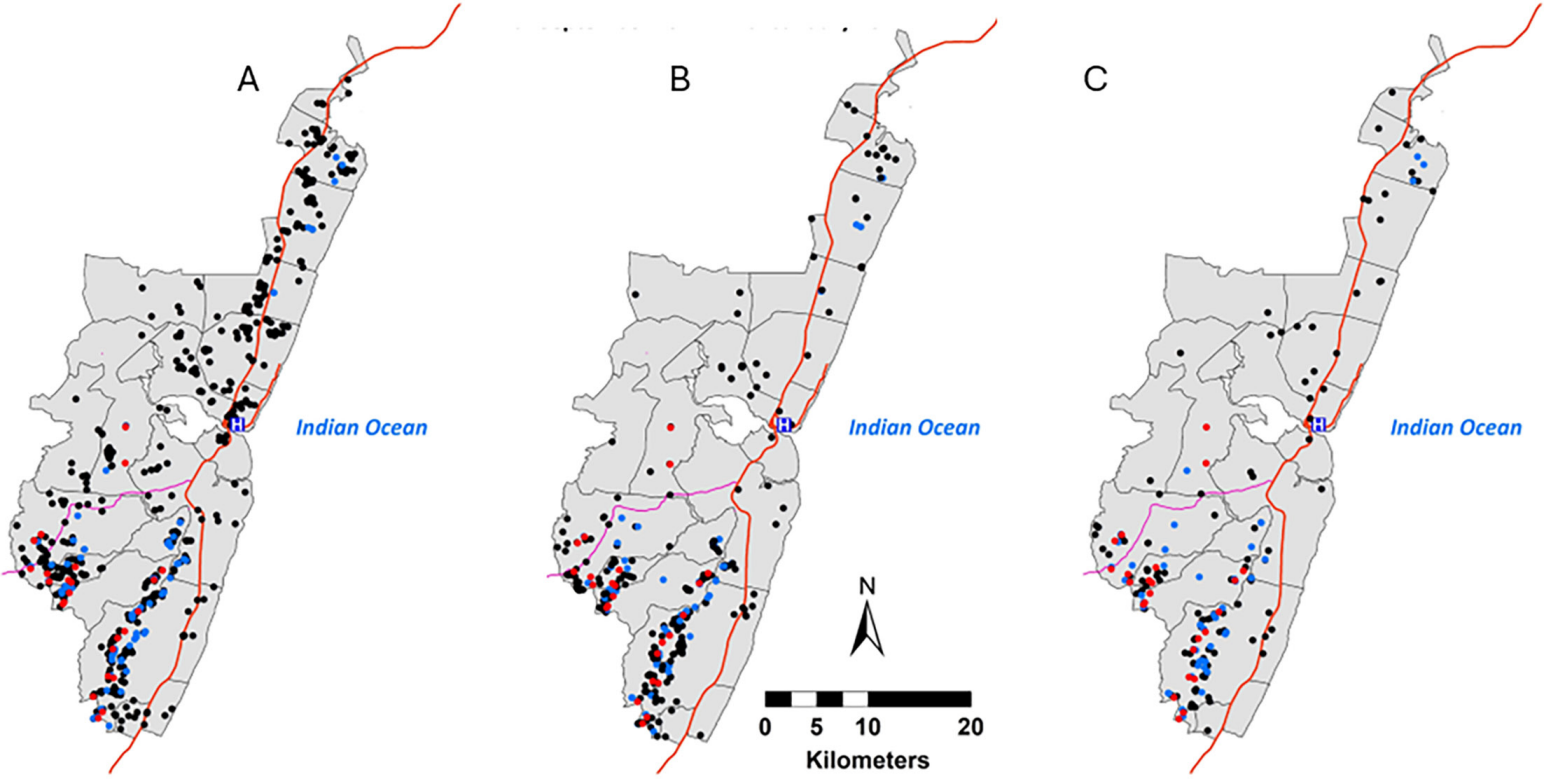
Distribution of tungiasis affected households by KHDSS rounds. (**A**) Round 1: 4 May–23 September 2021, (**B**) Round 2: 24 September 2021–14 January 2022, (**C**) Round 3: 15 January–4 May 2022. Each red dot represents a household reporting at least one case in all three rounds. A blue dot represents a household reporting at least one case in at least two rounds. A black dot represents a household reporting at least one case in only one round. The Kilifi County hospital (blue H), major roads (red lines) and administrative location boundaries (grey lines) are marked. KHDSS, Kilifi Health and Demographic Surveillance System; tungiasis, sand flea disease.

**Figure 3:**
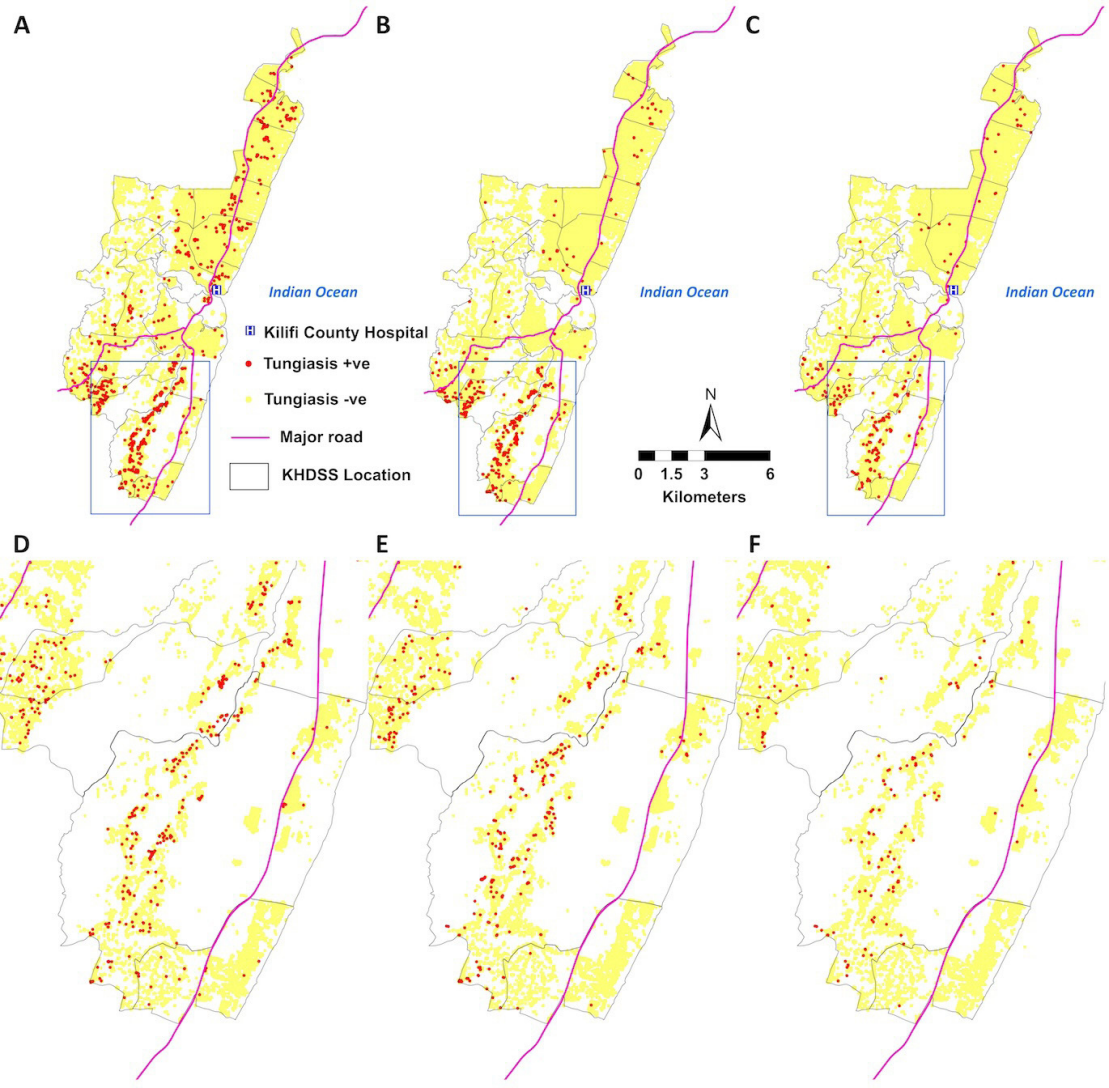
Distribution of households with (red dots) and without (yellow dots) a tungiasis case in the KHDSS. (**A and D**): round 1, (**B and E**): round 2, (**C and F**): round 3. (**D, E and F**) Show the southern section of the KHDSS at higher magnification for each of the respective survey rounds. The Kilifi County hospital (blue H), major roads (red lines) and administrative location boundaries (grey lines) are marked. KHDSS, Kilifi Health and Demographic Surveillance System; tungiasis, sand flea disease.

All administrative locations within the KHDSS area had at least one household having a case of tungiasis infection in all the three rounds (figure 2), but most affected households were in the southern section of the KHDSS. Household infection rarely persisted over time, with only 25 (1.8% of all affected households) reporting a case in all three rounds (figure 2), while 90.2% (1241) of affected households had a case in only one of the three rounds. The 25 households that consistently reported a case in all three surveys were only in the southern section of the KHDSS. Figure 3 shows that households with cases are frequently surrounded by unaffected households

### Risk factors for household reporting at least one case of tungiasis

All explanatory variables were significantly associated with having a case of tungiasis in the household in the univariable analyses (online supplemental table S4). In the final multivariable model, higher odds of having a case of tungiasis were associated with an earthen floor, an unimproved wall and rural locations. In addition, the higher the number of children under 15 years in a household and the higher the population density within 1 km of a household, the higher the odds of having tungiasis (table 2). Environmental variables positively associated with having a tungiasis case in the household were rainfall (average for the months of the survey round), EVI, LST, long-term aridity and altitudes over 50 m. However, there was a negative association with LULC categories of shrub land and built-up area. Of the soil properties, organic carbon content was positively associated, while aluminium content in the soil was negatively associated with having a case of tungiasis. Online supplemental figure S2 in the illustrates the geographical distribution of households reporting a case relative to the environmental variables. Having a case of tungiasis in the household varied significantly with administrative location and decreased over the survey rounds. Roof materials, NTL, soil pH and soil texture had no significant associations with tungiasis infection.

**Table 2:** Factors associated with having at least one tungiasis case in a household over three survey rounds*

Variable	Category	Households	Model 1			Model 2		
		N† (%)	AOR‡	95% CI**	P value§	AOR	95% CI	P value
Roof	Improved	47 323 (57.7)	1					
	Unimproved	34 667 (42.3)	1.1	0.92 to 1.25	0.359			
Floor	Non-earthen	21 630 (26.4)	1			1		
	Earthen	60 360 (73.6)	1.9	1.45 to 2.55	<0.001	1.9	1.47 to 2.57	<0.001
Wall	Improved	28 858 (35.2)	1			1		
	Unimproved	53 132 (64.8)	1.3	1.06 to 1.65	0.012	1.4	1.10 to 1.66	0.004
Household location	Urban	10 249 (12.5)	1			1		
	Rural	71 741 (87.5)	2.8	1.31 to 5.94	0.008	3.1	1.53 to 6.22	0.002
Population density	–	63 (36–99)	1.3	1.13 to 1.52	<0.001	1.3	1.15 to 1.54	<0.001
Number of children (<15 years)	–	1 (0–10)	1.2	1.15 to 1.25	<0.001	1.2	1.14 to 1.24	<0.001
Number adults>60 years	–	1 (0–5)	1.1	0.99 to 1.27	0.076			
Rainfall (mm)	–	5 (2–12)¶	3.2	2.45 to 4.28	<0.001	3.2	2.43 to 4.21	<0.001
EVI	–	8.6 (7.9–9.2)¶	1.3	1.17 to 1.55	<0.001	1.3	1.14 to 1.48	<0.001
LST (K)	–	30.7 (30.1–30.9)¶	1.2	1.09 to 1.22	<0.001	1.2	1.09 to 1.21	<0.001
Aridity	–	9.2 (8.2–9.7)¶	1.4	1.12 to 1.81	0.004	1.4	1.12 to 1.80	0.003
NTL	–	0 (0–0)¶	0.9	0.80 to 1.18	0.758			
Altitude (m asl)	0	39 758 (48.5)	1			1		
	0–50	19 166 (23.4)	1.1	0.80 to 1.59	0.481	1.1	0.79 to 1.52	0.585
	51–100	10 134 (12.4)	2.5	1.68 to 3.78	<0.001	2.5	1.68 to 3.68	<0.001
	101–255	12 932 (15.8)	2.4	1.52 to 3.80	<0.001	2.3	1.47 to 3.59	<0.001
LULC	Tree Cover	46 725 (56.9)	1			1		
	Built-up area	16 123 (19.7)	0.6	0.46 to 0.82	0.001	0.6	0.45 to 0.80	0.001
	Shrubland	11 290 (13.8)	0.5	0.35 to 0.60	<0.001	0.5	0.35 to 0.60	<0.001
	Grassland	6924 (8.4)	0.8	0.59 to 1.03	0.080	0.8	0.59 to 1.04	0.086
	Cropland	928 (1.0)	0.8	0.36 to 1.86	0.637	0.8	0.36 to 1.85	0.631
Soil pH	–	6.4 (6.2–6.6)¶	1.03	0.98 to 1.07	0.213			
Organic carbon content (g/kg)	–	1.5 (1.1–1.9)¶	2.9	2.13 to 4.04	<0.001	2.8	2.08 to 3.83	<0.001
Aluminium content (10 mg/kg)	–	62 (59–66)¶	0.9	0.995 to 0.999	0.044	0.9	0.995 to 0.999	0.034
Iron content (10 mg/kg)	–	13 (12–14)¶	0.9	0.99 to 1.01	0.885			
Soil texture	Sandy clay loam	59 970 (73.1)	1					
	Sandy loam	22 020 (26.9)	0.9	0.66 to 1.12	0.270			
Location	Banda ra salam	2771 (3.4)	1			1		
	Junju	9285 (11.3)	0.9	0.75 to 1.31	0.930	0.9	0.28 to 0.60	<0.001
	Chasimba	5374 (6.6)	0.4	0.28 to 0.54	<0.001	0.4	0.29 to 0.75	0.002
	Ziani	3940 (4.8)	0.5	0.37 to 0.69	<0.001	0.5	0.75 to 1.31	0.897
	Matsangoni	4801 (5.9)	0.7	0.39 to 1.37	0.332	0.8	0.16 to 0.36	<0.001
	Kilifi township	13 805 (16.8)	0.4	0.27 to 0.59	<0.001	0.4	0.27 to 0.52	<0.001
	Tezo	10 129 (12.4)	0.5	0.30 to 0.80	0.004	0.5	0.23 to 0.79	0.007
	Roka	5294 (6.5)	0.4	0.21 to 0.76	0.005	0.4	0.24 to 0.76	0.004
	Ngerenya	5231 (6.4)	0.4	0.22 to 0.77	0.005	0.4	0.44 to 1.42	0.438
	Takaungu mavueni	8809 (10.7)	0.2	0.15 to 0.35	<0.001	0.2	0.14 to 0.55	<0.001
	Kauma	2582 (3.2)	0.5	0.33 to 0.82	0.005	0.5	0.36 to 0.68	<0.001
	Mtwapa	3971 (4.8)	0.3	0.14 to 0.58	<0.001	0.3	0.05 to 0.19	<0.001
	Sokoke	2618 (3.2)	0.1	0.05 to 0.20	<0.001	0.1	0.34 to 0.82	0.005
	Jaribuni	1384 (1.7)	0.2	0.11 to 0.43	<0.001	0.2	0.08 to 0.53	0.001
	Gede	1996 (2.4)	0.2	0.07 to 0.50	<0.001	0.2	0.11 to 0.43	<0.001
Survey round	1	–	1			1		
	2	–	0.4	0.26 to 0.53	<0.001	0.4	0.23 to 0.48	<0.001
	3	–	0.3	0.19 to 0.51	<0.001	0.3	0.17 to 0.43	<0.001

* Multilevel univariable analysis is presented in online supplemental table S3.

† Total number of households in all three rounds, except for the variable ‘survey round’.

‡ Adjusted OR.

§ Associated p value.

¶ Median and IQRs, units of these variables are 10 times larger than presented.

** CI.

EVI, Enhanced Vegetation Index; LST, land surface temperature; LULC, Land Use Land Cover; NTL, night-time light; tungiasis, sand flea disease.

The final model showed a reasonable fit, with a Pseudo R² of 0.326 and 0.239 variability in the outcome. The LR test (p<0.001) indicated that the model significantly improved on the null model. In total, 32.6% of the variability in the outcome was explained by environmental variables, administrative location and survey round, and 24% was explained by environmental factors alone. Prominent environmental factors were altitude (3.6%), carbon content (3.5%), rainfall (2.5%) and aridity (2.4%) with respective pseudo R^2^ (online supplemental figure S3).

## Discussion

This study was the largest household survey of tungiasis to date, that included detailed geospatial data and environmental variables. We found the prevalence of tungiasis decreased from 1.1% between May and September 2021 to 0.24% between January and May 2022 and its spatial distribution was heterogenous, being clustered mostly in the southern areas of the survey zone. The households with cases were heterogenous over time, with only 1.8% of affected households being persistently infected and 90.2% being infected in only one survey round. The temporal and spatial distribution was partly explained by the house floor and wall type, number of children under 15 years of age in a household, population density and several environmental factors, which accounted for 23.9% of the variability.

The prevalence found during the first survey round was very similar to the national prevalence of 1.35% reported from a randomised survey of school children in nine counties of Kenya conducted at the same time.^6^ Only one other large-scale household survey has ever been conducted for tungiasis and that was of 7925 households in the routine HDSS survey in Kwale County bordering the south of the current study area, in 2011.^19^ The household prevalence there was 3.4%, higher than any of the survey rounds in our Kilifi survey. This difference in prevalence could be the result of differences in the year of the survey, altitude, climate, cultures and proximity to wildlife parks.

Although the household prevalence in the KHDSS is low, and tungiasis rarely results in mortality, the symptomatic and social consequences of the infection are substantial. Chronic pain and itching lead to difficulty walking, inability to sleep, attend school or work, concentrate on work and, as a result, lower examination results.^20^ People with tungiasis also experience stigma and discrimination.^21^ Even if an individual is infected for only 3 months of a year, that could be the entirety of a school term, and although we were not able to continue the study for another year, it is possible the same families are infected every year.

The multivariable analyses found a strong association between tungiasis and unimproved floors and walls which are both linked to poverty and are well-established risk factors for tungiasis.^22^,^24^ It has been demonstrated that the off-host cycle mostly takes place in the soil floors of sleeping rooms of infected people.^15 25^ Since 72.9% of all households in the survey had an earthen floor, but tungiasis was rare among them, having an earthen floor is not predictive of infection, only a greater risk, while a sealed floor is protective.

We found a strong association with the number of children under 15 years in a household and population density. One past study included the density of the rural poor population at a 5 km resolution in niche modelling for tungiasis in sub-Saharan Africa and found it to be a major contributor to the model.^11^ Other studies have found that the more people sleep in a house, the higher the odds that any one of them will be infected.^19 24 26^ These associations are not unexpected since the prevalence and transmission of any disease depend on susceptible hosts encountering the parasite (adult female sand fleas). The higher the density of host populations, the more of them may be exposed to soil infested with *Tunga penetrans*. Children under 15 years have been shown to have the highest prevalence and odds of infection.^24^ While adults over 60 years have been shown to have higher prevalence and infection intensity than younger adults,^24 27^ there was no association with the presence nor the number of adults over 60 in the current study.

The environmental conditions required for the *Tunga penetrans* larvae to thrive have been reported to be between 22.0°C and 31.2°C and at a humidity from 51.4% to 95%.^15^ We found both LST and rainfall to be associated with the presence of tungiasis even within the narrow range that occurred in the KHDSS during the study period. The LST median was 30.7°C with an IQR of 30.1–30.9°C, and the rainfall median was 50 mm with an IQR of 20–120 mm. We did not collect data on humidity, but this varies between 75% and 82% in Kilifi County.^28^

Even though vegetation cover is linked to rainfall and temperature, the EVI and LULC were both also independently associated with tungiasis infection, being highest in areas with tree cover and high EVI and lowest in built-up areas, shrubland and low EVI. Trees might provide shade and keep daytime temperatures lower and humidity higher, even inside houses if they are shaded, while in built-up areas and shrubland the ground is likely exposed to the direct sun for most of the day and temperatures would be higher and humidity lower and inhospitable to the survival and development of the off-host stages. While most transmission occurs indoors,^25^ these factors would determine whether any can take place outdoors. Only one past study has compared exposed and shaded outdoor areas, and larvae were only found in positions not directly exposed to the sun at any time in the day.^15^

We included macrolevel soil characteristics in the models since affected communities regularly associate infection with the colour of the soil (anecdotal reports) which reflects its chemical composition, and organic carbon represents the organic matter that larvae require for nutrition. Indeed, we found a positive association with organic carbon content, but a negative association with aluminium content. Aluminium is one of the most common components of inorganic soil particles.^29^ In its solid forms such as silicates and oxides, it is inert, but as chlorides, nitrates and sulfates, it is soluble in water under acidic conditions and is toxic to mammals, plants,^29^ nematodes^30^ and insects.^31^ It is conceivable that higher aluminium content is toxic to the sand flea larvae. However, in the study in the Kwale HDSS, there was a positive association with aluminium content but not organic carbon.^19^ Neither of our respective studies found associations with soil pH, iron content of the soil or the soil texture. Further geospatial studies are needed that incorporate a wider range in these factors as well as microlevel assessments of soils inside houses and laboratory bioassays, to determine whether soil composition does play a role in transmission and disease distribution.

While the altitude of the KHDSS area only ranges from sea level to 255 m, we found a positive association of tungiasis with the ordinal altitude variable. Households above 50 m asl had an almost three times higher odds of having at least one case of tungiasis than those at sea level. This agrees with the findings of Hyuga *et al* who also found a positive correlation with altitude.^19^ While this finding was independent of the climate and soil factors (that may vary with altitude) in the model, it may be the result of different sociodemographic and behavioural factors in these areas that were not included here.

The survey round remained statistically significant after adjusting for environmental factors in the final multivariable model, with the households having a lower odds of infection in rounds 2 and 3, even when adjusted for all the other variables. Since VIF also indicated no collinearity between variables, this suggests that those variables that would have changed with time, such as rainfall and LST, did not fully explain the change in prevalence over time nor the spatial distribution of affected households. Furthermore, the variables included in the final model accounted for only 23.9% of the variability in tungiasis distribution, indicating other unmeasured factors are important. Other studies that have examined non-environmental factors have demonstrated that socio-economic, family and behavioural aspects play a key role in determining tungiasis infection, including personal hygiene behaviour,^24 32^ shoe-wearing,^23 33^ defecation practice^22 23^ and caregiver education.^34 35^ It is possible that some behavioural factors change over time and may be linked to the changing seasons such as at planting or harvest time, which could put people at more risk. At harvest time, families may have higher incomes and be better able to afford more water and soap for washing. There is also the possibility that interventions unknown to the authors were implemented by the county public health officers or their partners. Lastly, having field officers from KEMRI-Wellcome Trust in the community asking about tungiasis may have raised awareness of the disease so that people started paying more attention to it and taking action to control it themselves.

The main limitation of this study was that the outcome variable depended on a household representative reporting the presence of a current tungiasis case accurately, rather than having household members examined. This could lead to inaccurate reporting if the respondent was not aware of any infection in the family or would rather conceal an infection due to their own feelings of shame and self-stigma and expectation of discrimination which are often experienced by infected individuals.^21^ They may also wish to conceal infection out of fear of any cases being given treatment which may be painful, such as mechanical extraction.^36^ This limitation is mitigated by several factors. First, residents of endemic areas tend to be very good at self-diagnosis and identifying infection in others,^37 38^ although they may not differentiate current infection with live fleas from past infection which may leave long-lasting, visible disfigurement. Second, the households had been participating in the KHDSS for 20 years and a good relationship had been built between KEMRI researchers, field officers and community members through the continual visits, the regular meetings of community representatives and the school engagement programme.^39 40^ Another factor is the community, through their representatives, who had repeatedly requested KEMRI conduct research on tungiasis, indicating commitment to supporting surveillance. Lastly, two school-based surveys for tungiasis which used direct observation^6^ (and one by the ministry of health but unpublished) conducted in 2021 and 2022, reported similar county level prevalences for Kilifi of 0.58% and 1.6%, respectively, and reported the same clustering of cases in the southern areas of the KHDSS zone.

Another limitation of the study is that the KHDSS covers a relatively small geographical area of 891 km^2^ with a limited range in altitude, temperature, humidity, rainfall, vegetation and soil types. The household factors that could be tested were also a limitation for the study, being restricted to what was already available in the standard KHDSS survey instruments.

## Conclusion

This study using the routine KHDSS and environmental factors in publicly available geo-referenced databases has identified environmental factors that were associated with tungiasis, accounting for 21.9% of the variability in distribution of cases. This confirms the potential for niche modelling to guide tungiasis control activities. To have generalisability across Kenya and/or Africa will require surveys with a larger range in all environmental variables and in further geographical settings. Control activities, socio-economic status and behavioural factors also play a role, so that geospatial prediction of tungiasis distribution can indicate an area that may be suitable for transmission but cannot definitively indicate infection. Control programmes will therefore benefit from a combination of predicted transmission areas followed by confirmatory targeted local surveys.

## Supplementary material

10.1136/bmjgh-2025-020057 online supplemental file 1

10.1136/bmjgh-2025-020057 online supplemental file 2

## Data Availability

Data are available in a public, open access repository.
