# Supplementary material for: Prevalence, spatial and temporal distribution of tungiasis in the Kilifi Health and Demographic Surveillance System (KHDSS) in Kenya
Source: BMJ Glob Health. 2026 Mar 4;11(3):e020057. doi: 10.1136/bmjgh-2025-020057 (PMC12970124; doi:10.1136/bmjgh-2025-020057)
Supplement: online supplemental file 1 [file bmjgh-11-3-s001.docx]

**Supplementary materials**

**Prevalence, Spatial and Temporal Distribution of Tungiasis in the Kilifi Health and Demographic Surveillance System (KHDSS) in Kenya**

Nelson Ouma, MSc^1*^ · Samuel K. Muchiri, BSc^2^ · Christopher Nyundo, MSc^1^ · David Walumbe, MSc^1^· Amek Nyaguara, PhD^1^ · Marta Maia, PhD^1,3^ · Ifedayo Adetifa, PhD^1,4^ · Benedict Orindi, PhD^1^ · Phillip Bejon, PhD^1,3^ · Ulrike Fillinger, PhD^5^, ·Lynne Elson, PhD^1,3^

^1^Kenya Medical Research Institute (KEMRI)-Wellcome Trust, Kilifi, Kenya,

^2^Population and Health Impact Surveillance Group, KEMRI-Wellcome Trust, Nairobi, Kenya,

^3^Nuffield Department of Medicine, University of Oxford,

^4^London School of Hygiene & Tropical Medicine, Keppel Street, London, UK,

^5^International Centre for Insect Physiology and Ecology (icipe) Kenya.

**Contents**

[Table S1: Household and altitude categorization and definition of improved and unimproved variables 2](#_Toc205535594)

[Table S2: Environmental and Ecological data used in the analysis and its sources and details. 3](#_Toc205535595)

[Figure S1: Environmental, and soil covariates maps 4](#_Toc205535596)

[Figure S2: Environmental and soil covariate maps overlayed with households having at least one tungiasis case from survey round 2. 5](#_Toc205535597)

[Table S3: Distribution of households by each variable and tungiasis status 6](#_Toc205535598)

[Table S4: Univariable multilevel logistic regression for factors associated with having at least one tungiasis case in a household over three survey rounds. 9](#_Toc205535599)

[Figure S3: Pseudo R^2^ of each variable in the final model (model 2). 12](#_Toc205535600)

### Table S1: Household and altitude categorization and definition of improved and unimproved variables

| **Household variable** | **Improved** | **Unimproved** |
| --- | --- | --- |
| House type | Town house | Rural type |
|  | Courtyard house |  |
| Roof | Metal/Corrugated | Makuti |
|  | Concrete | Grass thatched |
|  | Tiled | Polythene |
|  | Asbestos | Other |
| Floor | Concrete | Wooden |
|  | Local stone tiles | Loose soil/sand/coral sand |
|  |  | Other |
| Wall | Brick/concrete/coral | Wooden |
|  |  | Mud stick |
|  |  | Other |
|  |  | Iron sheet |
|  |  | Grass/Makuti |
| Altitude (Meters) | 0 | |
|  | 1-50 | |
|  | 51-100 | |
|  | 101-255 | |

### Table S2: Environmental and Ecological data used in the analysis and its sources and details.

| **Covariate** | **Type** | **Temporal Extents** | **Spatial Resolution** | **Source** |
| --- | --- | --- | --- | --- |
| Land use land cover (LULC) | Temporal | 2021 (Annual) | 10m | [ESA - https://worldcover2020.esa.int/download](https://eur01.safelinks.protection.outlook.com/?url=https%3A%2F%2Fworldcover2020.esa.int%2Fdownload&data=05%7C02%7CNOuma%40kemri-wellcome.org%7C58df575b55ec40eeb60b08dbff8c7ceb%7Ca5c0a820c8874727ac66403237d8c389%7C0%7C0%7C638384751075860383%7CUnknown%7CTWFpbGZsb3d8eyJWIjoiMC4wLjAwMDAiLCJQIjoiV2luMzIiLCJBTiI6Ik1haWwiLCJXVCI6Mn0%3D%7C3000%7C%7C%7C&sdata=823btdaQ7Fpk%2BGupp8bYYNw7%2BzuaAwxrI1ydsJaboHM%3D&reserved=0) |
| Vegetation cover: Enhance vegetation index (EVI) | Temporal | 2021 – 2022 (16-day) | 250m | [MODIS - https://modis.gsfc.nasa.gov/data/dataprod/mod13.php](https://eur01.safelinks.protection.outlook.com/?url=https%3A%2F%2Fmodis.gsfc.nasa.gov%2Fdata%2Fdataprod%2Fmod13.php&data=05%7C02%7CNOuma%40kemri-wellcome.org%7C58df575b55ec40eeb60b08dbff8c7ceb%7Ca5c0a820c8874727ac66403237d8c389%7C0%7C0%7C638384751075860383%7CUnknown%7CTWFpbGZsb3d8eyJWIjoiMC4wLjAwMDAiLCJQIjoiV2luMzIiLCJBTiI6Ik1haWwiLCJXVCI6Mn0%3D%7C3000%7C%7C%7C&sdata=2DNd3KtqePOIlEJp1FM5d2OPO1d19%2B1wa1zsRlj1J%2BU%3D&reserved=0) |
| Mean monthly Rainfall | Temporal | 2021 – 2022 | 0.05 deg (~ 5 x 5 km) resampled to 500 x 500m | [CHIRPS - https://data.chc.ucsb.edu/products/CHIRPS-2.0/global_annual/tifs/](https://eur01.safelinks.protection.outlook.com/?url=https%3A%2F%2Fdata.chc.ucsb.edu%2Fproducts%2FCHIRPS-2.0%2Fglobal_annual%2Ftifs%2F&data=05%7C02%7CNOuma%40kemri-wellcome.org%7C58df575b55ec40eeb60b08dbff8c7ceb%7Ca5c0a820c8874727ac66403237d8c389%7C0%7C0%7C638384751075860383%7CUnknown%7CTWFpbGZsb3d8eyJWIjoiMC4wLjAwMDAiLCJQIjoiV2luMzIiLCJBTiI6Ik1haWwiLCJXVCI6Mn0%3D%7C3000%7C%7C%7C&sdata=fPTi2%2FUbzfDbgyRWMB3kDqAC%2BKQob7N8DI8ZNEXXR%2FY%3D&reserved=0) |
| Land surface temperature (LST) | Temporal | 2021 – 2022 (16-day) | 500m | [MODIS - https://modis.gsfc.nasa.gov/data/dataprod/mod11.php](https://eur01.safelinks.protection.outlook.com/?url=https%3A%2F%2Fmodis.gsfc.nasa.gov%2Fdata%2Fdataprod%2Fmod11.php&data=05%7C02%7CNOuma%40kemri-wellcome.org%7C58df575b55ec40eeb60b08dbff8c7ceb%7Ca5c0a820c8874727ac66403237d8c389%7C0%7C0%7C638384751075860383%7CUnknown%7CTWFpbGZsb3d8eyJWIjoiMC4wLjAwMDAiLCJQIjoiV2luMzIiLCJBTiI6Ik1haWwiLCJXVCI6Mn0%3D%7C3000%7C%7C%7C&sdata=CZErLwCPknrRGatQ2dWFkED7jKaqp9xF0kCEUqgOAsE%3D&reserved=0) |
| Aridity | Long-Term Average | 1970 – 2000 (Long-term average) | 1km | [CGIAR-CSI GeoPortal - https://cgiarcsi.community/data/global-aridity-and-pet-database/](https://eur01.safelinks.protection.outlook.com/?url=https%3A%2F%2Fcgiarcsi.community%2Fdata%2Fglobal-aridity-and-pet-database%2F&data=05%7C02%7CNOuma%40kemri-wellcome.org%7C58df575b55ec40eeb60b08dbff8c7ceb%7Ca5c0a820c8874727ac66403237d8c389%7C0%7C0%7C638384751075860383%7CUnknown%7CTWFpbGZsb3d8eyJWIjoiMC4wLjAwMDAiLCJQIjoiV2luMzIiLCJBTiI6Ik1haWwiLCJXVCI6Mn0%3D%7C3000%7C%7C%7C&sdata=7ICf534j9X2iJANae7ARPOEAR%2FgO%2BW8xKMNYbgjXPE8%3D&reserved=0) |
| Elevation | Long-Term Average | - | 30m | [MODIS - https://earthexplorer.usgs.gov/](https://eur01.safelinks.protection.outlook.com/?url=https%3A%2F%2Fearthexplorer.usgs.gov%2F&data=05%7C02%7CNOuma%40kemri-wellcome.org%7C58df575b55ec40eeb60b08dbff8c7ceb%7Ca5c0a820c8874727ac66403237d8c389%7C0%7C0%7C638384751076017584%7CUnknown%7CTWFpbGZsb3d8eyJWIjoiMC4wLjAwMDAiLCJQIjoiV2luMzIiLCJBTiI6Ik1haWwiLCJXVCI6Mn0%3D%7C3000%7C%7C%7C&sdata=XIcn5sSmZqdy3WTJ7jpKU7ds4gctpltrP%2BjO2a%2BCa1k%3D&reserved=0) |
| NTL | Temporal | 2021 (Annual) | 500m | [NOAA - https://ngdc.noaa.gov/eog/viirs.html](https://eur01.safelinks.protection.outlook.com/?url=https%3A%2F%2Fngdc.noaa.gov%2Feog%2Fviirs.html&data=05%7C02%7CNOuma%40kemri-wellcome.org%7C58df575b55ec40eeb60b08dbff8c7ceb%7Ca5c0a820c8874727ac66403237d8c389%7C0%7C0%7C638384751076017584%7CUnknown%7CTWFpbGZsb3d8eyJWIjoiMC4wLjAwMDAiLCJQIjoiV2luMzIiLCJBTiI6Ik1haWwiLCJXVCI6Mn0%3D%7C3000%7C%7C%7C&sdata=MokLNjINSM0KIUzqTF8EDHaVgcclDPyzjJLSjPljaCo%3D&reserved=0) |
| Soil pH | Long-Term Average | 1981 - 2016 | 250m | https://data.isric.org/geonetwork/srv/eng/catalog.search#/metadata/4c59ee58-a24e-4154-912e-0ff18395ac0d |
| Soil texture | Long-Term Average | 1982 - 2016 | 250m | https://data.isric.org/geonetwork/srv/eng/catalog.search#/metadata/f9a3a4e0-27a8-4acc-861f-26c112699c3e |
| Soil organic carbon content | Long-Term Average | 1983 - 2016 | 250m | https://data.isric.org/geonetwork/srv/eng/catalog.search#/metadata/076db4e8-11a9-4262-b6aacfa703a3c0af |
| Aluminium content in the soil | Long-Term Average | 1984 - 2016 | 250m | https://data.isric.org/geonetwork/srv/eng/catalog.search#/metadata/a36f7919-0d6e-4044-902c-64a74feade6b |
| Iron content in the soil | Long-Term Average | 1985 - 2016 | 250m | https://data.isric.org/geonetwork/srv/eng/catalog.search#/metadata/5cd5336c-2f45-4430-a9a8-312aa2095cb6 |

### Figure S1: Environmental, and soil covariates maps


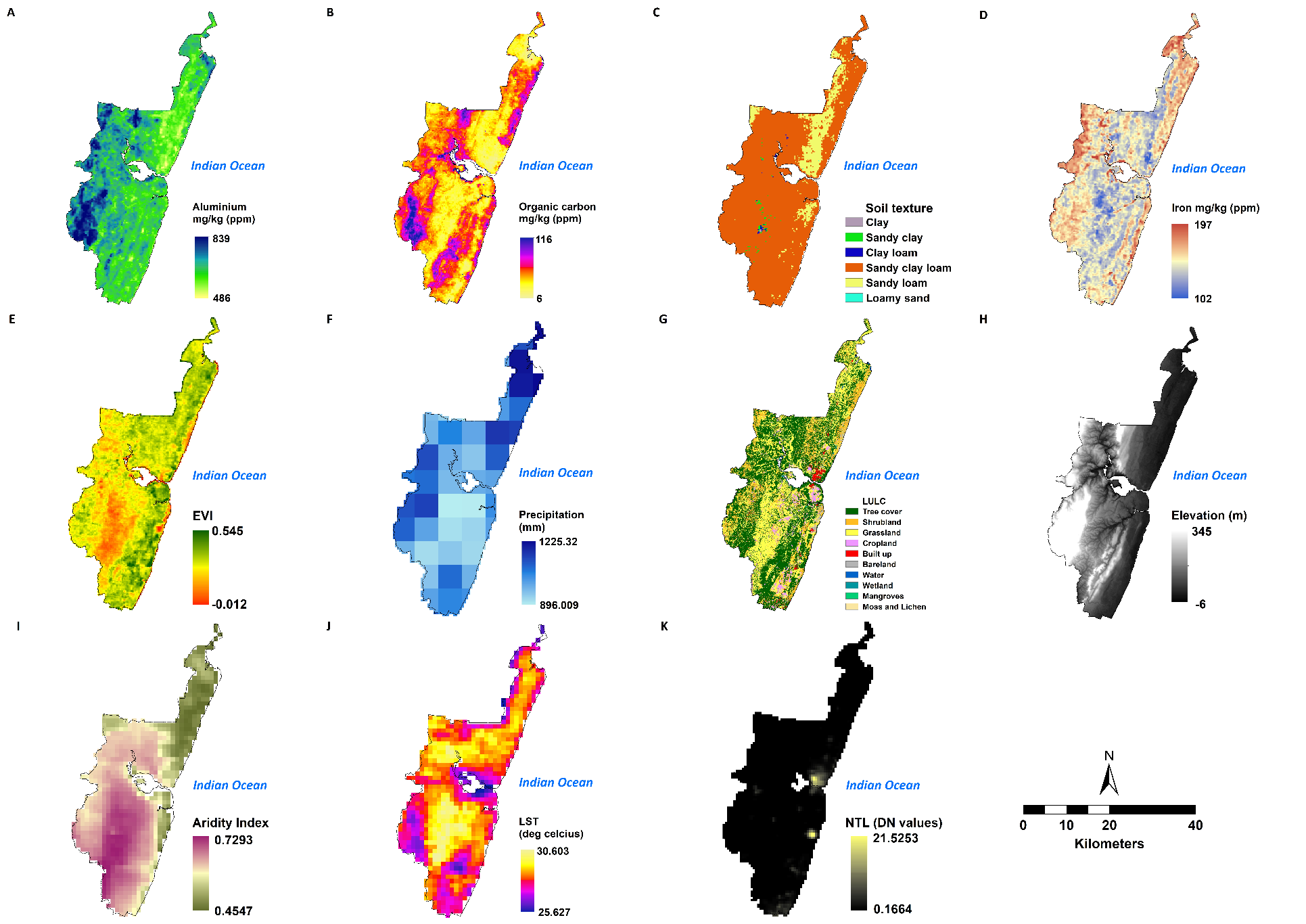


### Figure S2: Environmental and soil covariate maps overlayed with households having at least one tungiasis case from survey round 2.


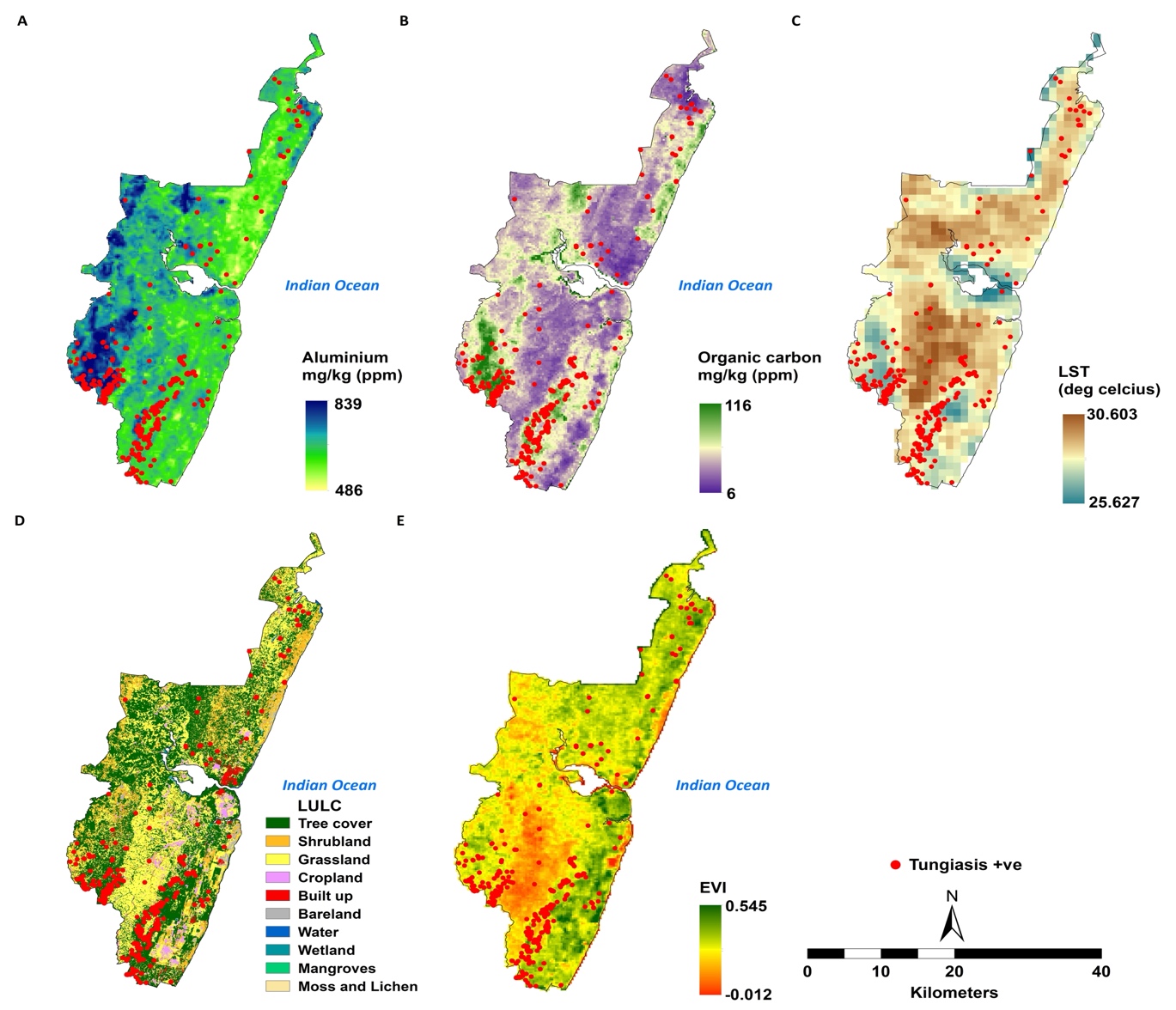


### Table S3: Distribution of households by each variable and tungiasis status

|  |  | All Households | Households with a tungiasis case | Households with NO case |
| --- | --- | --- | --- | --- |
| Variable | Category | N (%) | N (%) | N (%) |
| ***Household Characteristics*** |  |  |  |  |
| Roof | Improved | 47323 (57.7) | 627 (45.6) | 46696 (57.9) |
|  | Unimproved | 34667 (42.3) | 749 (54.4) | 33918 (42.1) |
| Floor | Non-earthen | 21630 (26.4) | 128 (9.3) | 21502 (26.7) |
|  | Earthen | 60360 (73.6) | 1248 (90.1) | 59112 (73.3) |
| Wall | Improved | 28858 (35.2) | 253 (18.4) | 28605 (35.5) |
|  | Unimproved | 53132 (64.8) | 1123 (81.6) | 52009 (64.5) |
| Household location | Urban | 10249 (12.5) | 28 (2.0) | 10221 (12.7) |
|  | Rural | 71741 (87.5) | 1348 (98.0) | 70393 (87.3) |
| House occupancy | 1 | 4666 (5.7) | 79 (0·56) | 4587 (5.7) |
|  | 2-5 | 23911 (29.2) | 354 (0·49) | 23557 (29.2) |
|  | 6-9 | 23532 (28.7) | 484 (0·69) | 23048 (28.6) |
|  | ≥10 | 29881 (36.4) | 608 (0·69) | 29273 (36.3) |
| Number of children <15 years | - | 1 (0 - 10)* | 1 (0 – 7) | 1 (0 - 10) |
| Number of adults >60 years | - | 1 (0 - 5)* | 1 (0 – 3) | 1 (0 - 5) |
| Presence of children <15 years | No | 43492 (53·1) | 615 (44·7) | 42877 (53.2) |
|  | Yes | 38498 (46·9) | 761 (55·3) | 37737 (46.8) |
| Presence adults >60 years | No | 65459 (79·8) | 1071 (77·8) | 64388 (79.9) |
|  | Yes | 16531 (20·2) | 305 (22·2) | 16226 (20.1) |
| Population density (/km^2^) | - | 63 (36-99)* | 72 (45-108) | 63 (36-99) |
| ***Environmental & Ecological Characteristics*** | | | | |
| Rainfall (mm) | - | 5 (2 − 12)* | 5 (3-12) | 4 (3 – 12) |
| EVI | - | 8·6 (7·9 − 9·2)* | 8.4 (7.9 – 9.1) | 8·7 (7·9 − 9·3) |
| LST (K) | - | 30·7(30·1 − 30·9)* | 30.3 (29.9 – 30.9) | 30·7(30·1 − 30·9) |
| Aridity | - | 9·2 (8·2 − 9·7)* | 9.8 (8.9 – 10.1) | 9·2 (8·2 − 9·7 |
| NTL | - | 0 (0 − 0)* | 0 (0 − 0) | 0 (0 − 0) |
| Altitude (m asl) | Sea level | 39758 (48.5) | 357 (25.9) | 39401 (48.9) |
|  | 0-50 | 19166 (23.4) | 177 (12.9) | 18989 (23.6) |
|  | 51-100 | 10134 (12.4) | 404 (29.4) | 9730 (12.1) |
|  | 101-255 | 12932 (15.8) | 438 (31.8) | 12494 (15.5) |
| LULC | Tree Cover | 46725 (56.9) | 1095 (79.6) | 45630 (56.6) |
|  | Built-up area | 16123 (19.7) | 91 (6.6) | 16032 (19.9) |
|  | Shrubland | 11290 (13.8) | 98 (7.1) | 11192 (13.9) |
|  | Grassland | 6924 (8.4) | 84 (6.1) | 6840 (8.5) |
|  | Cropland | 928 (1.0) | 8 (0.6) | 920 (1.1) |
| ***Soil components and structure*** | | | |  |
| Soil pH | - | 6·4 (6·2-6·6)* | 6.3 (6.2 – 6.4) | 6·4 (6·2-6·6) |
| Organic carbon content (g/kg) | - | 1·5 (1·1-1·9)* | 1.9 (1.6 – 2.5) | 1·5 (1·1-1·9) |
| Aluminum content (10mg/kg) | - | 62 (59-66)* | 64 (62 – 68) | 62 (59-65) |
| Iron content (10mg/kg) | - | 13 (12-14)* | 13 (12 -14) | 13 (12-14) |
| Soil texture | Sandy clay loam | 59970 (73.1) | 1199 (87.1) | 58771 (72.9) |
|  | Sandy loam | 22020 (26.9) | 177 (12.9) | 21843 (27.1) |
| Location | Banda ra salam | 2771 (3.4) | 166 (12.1) | 2605 (3.2) |
|  | Junju | 9285 (11.3) | 380 (27.6) | 8905 (11.0) |
|  | Chasimba | 5374 (6.6) | 135 (9.8) | 5239 (6.5) |
|  | Ziani | 3940 (4.8) | 132 (9.6) | 3808 (4.7) |
|  | Matsangoni | 4801 (5.9) | 91 (6.6) | 4710 (5.8) |
|  | Kilifi township | 13805 (16.8) | 90 (6.5) | 13715 (17.0) |
|  | Tezo | 10129 (12.4) | 89 (6.4) | 10040 (12.5) |
|  | Roka | 5294 (6.5) | 78 (5.7) | 5216 (6.5) |
|  | Ngerenya | 5231 (6.4) | 64 (4.7) | 5167 (6.4) |
|  | Takaungu mavueni | 8809 (10.7) | 60 (4.4) | 8749 (10.9) |
|  | Kauma | 2582 (3.2) | 39 (2.8) | 2543 (3.2) |
|  | Mtwapa | 3971 (4.8) | 19 (1.4) | 3952 (4.9) |
|  | Sokoke | 2618 (3.2) | 11 (0.8) | 2607 (3.2) |
|  | Jaribuni | 1384 (1.7) | 12 (0.9) | 1372 (1.7) |
|  | Gede | 1996 (2.4) | 10 (0.7) | 1986 (2.5) |
| Survey Round | 1 | 81990 (91.9) | 869 (63.2) | 81121 (98.9) |
|  | 2 | 81990 (90.8) | 340 (24.7) | 81650 (99.6) |
|  | 3 | 81990 (89.9) | 167 (12.1) | 81823 (99.8) |

### Table S4: Univariable multilevel logistic regression for factors associated with having at least one tungiasis case in a household over three survey rounds.

Household was used as a random effect.

|  |  | Households | Households with a tungiasis case | Univariable model | | |
| --- | --- | --- | --- | --- | --- | --- |
| Variable | Category | N (%) | N (%) | OR | 95% CI | *p* |
| ***Household Characteristics*** | | | | | | |
| Roof | Improved | 47323 (57.7) | 627 (45.6) | 1 |  |  |
|  | Unimproved | 34667 (42.3) | 749 (54.4) | 1·6 | 1·48 −1·81 | <0·001 |
| Floor | Non-earthen | 21630 (26.4) | 128 (9.3) | 1 |  |  |
|  | Earthen | 60360 (73.6) | 1248 (90.1) | 3·8 | 3·23 − 4·62 | <0·001 |
| Wall | Improved | 28858 (35.2) | 253 (18.4) | 1 |  |  |
|  | Unimproved | 53132 (64.8) | 1123 (81.6) | 2·5 | 2·18 − 2·83 | <0·001 |
| Household location | Urban | 10249 (12.5) | 28 (2.0) | 1 |  |  |
|  | Rural | 71741 (87.5) | 1348 (98.0) | 2·0 | 1·67 − 2·42 | <0·001 |
| House occupancy | 1 | 13998(5·7) | 79 (0·56) | 1 |  |  |
|  | 2-5 | 71733 (29·4) | 354 (0·49) | 0·87 | 0·68 − 1·11 | 0·280 |
|  | 6-9 | 70596 (28·9) | 484 (0·69) | 1·22 | 0·95 − 1·54 | 0·108 |
|  | ≥10 | 87900 (35·9) | 608 (0·69) | 1·23 | 0·97 − 1·55 | 0·088 |
| Number of children <15 years | - | 1 (0 - 10)* | - | 1·2 | 1.16 − 1.23 | <0.001 |
| Number of adults >60 years | - | 1 (0 - 5)* | - | 1·1 | 1.01 − 1.22 | 0.020 |
| Presence of children <15 years | No | 43492 (53·1) | 615 (44·7) | 1 |  |  |
|  | Yes | 38498 (46·9) | 761 (55·3) | 1·5 | 1.33 − 1.64 | 0.005 |
| Presence adults >60 years | No | 65459 (79·8) | 1071 (77·8) | 1 |  |  |
|  | Yes | 16531 (20·2) | 305 (22·2) | 1·2 | 1.04 − 1.32 | 0.010 |
| Population density (/km^2^) | - | 63 (36-99)* | - | 1·13 | 1.03 − 1.24 | 0.009 |
| ***Environmental & Ecological Characteristics*** | | | | | | |
| Rainfall (mm) | - | 5 (2 − 12)* | - | 1·9 | 1·75 − 1·98 | <0·001 |
| EVI | - | 8·6 (7·9 − 9·2)* | - | 0·8 | 0·79 − 0·87 | <0·001 |
| LST (K) | - | 30·7(30·1 − 30·9)* | - | 0·9 | 0·89 − 0·92 | <0·001 |
| Aridity | - | 9·2 (8·2 − 9·7)* | - | 1·8 | 1·72 − 1·96 | <0·001 |
| NTL | - | 0 (0 − 0)* | - | 0·5 | 0·48 − 0·58 | <0·001 |
| Altitude (m asl) | Sea level | 39758 (48.5) | 357 (25.9) | 1 |  |  |
|  | 0-50 | 19166 (23.4) | 177 (12.9) | 1·1 | 0·94 − 1·33 | 0·201 |
|  | 51-100 | 10134 (12.4) | 404 (29.4) | 5·0 | 4·40 − 5·79 | <0·001 |
|  | 101-255 | 12932 (15.8) | 438 (31.8) | 4·3 | 3·79 − 4·97 | <0·001 |
| LULC | Tree Cover | 46725 (56.9) | 1095 (79.6) | 1 |  |  |
|  | Built-up area | 16123 (19.7) | 91 (6.6) | 0.2 | 0.19 − 0.29 | <0.001 |
|  | Shrubland | 11290 (13.8) | 98 (7.1) | 0.4 | 0.29 − 0.43 | <0.001 |
|  | Grassland | 6924 (8.4) | 84 (6.1) | 0.5 | 0.40 − 0.61 | <0.001 |
|  | Cropland | 928 (1.0) | 8 (0.6) | 0.4 | 0.18 − 0.72 | 0.004 |
| ***Soil components and structure*** | | | | | | |
| Soil pH | - | 6·4 (6·2-6·6)* | - | 0·8 | 0·83 − 0·87 | <0·001 |
| Organic carbon content (g/kg) | - | 1·5 (1·1-1·9)* | - | 1·03 | 1·02 − 1·03 | <0·001 |
| Aluminum content (10mg/kg) | - | 62 (59-66)* | - | 1·01 | 1·0 − 1·01 | <0·001 |
| Iron content (10mg/kg) | - | 13 (12-14)* | - | 1·01 | 1·0 − 1·01 | 0·015 |
| Soil texture | Sandy clay loam | 59970 (73.1) | 1199 (87.1) | 1 |  |  |
|  | Sandy loam | 22020 (26.9) | 177 (12.9) | 0·4 | 0·45 − 0·65 | <0·001 |
| Location | Banda ra salam | 2771 (3.4) | 166 (12.1) | 1 |  |  |
|  | Junju | 9285 (11.3) | 380 (27.6 | 0.7 | 0.58 − 0.81 | <0.001 |
|  | Chasimba | 5374 (6.6) | 135 (9.8) | 0.4 | 0.33 − 0.50 | <0.001 |
|  | Ziani | 3940 (4.8) | 132 (9.6) | 0.5 | 0.43 − 0.66 | <0.001 |
|  | Matsangoni | 4801 (5.9) | 91 (6.6) | 0.3 | 0.21 − 0.35 | <0.001 |
|  | Kilifi township | 13805 (16.8) | 90 (6.5) | 0.9 | 0.07 − 0.12 | <0.001 |
|  | Tezo | 10129 (12.4) | 89 (6.4) | 0.1 | 0.09 − 0.16 | <0.001 |
|  | Roka | 5294 (6.5) | 78 (5.7) | 0.2 | 0.16 − 0.27 | <0.001 |
|  | Ngerenya | 5231 (6.4) | 64 (4.7) | 0.2 | 0.13 − 0.23 | <0.001 |
|  | Takaungu mavueni | 8809 (10.7) | 60 (4.4) | 0.1 | 0.08 -− 0.13 | <0.001 |
|  | Kauma | 2582 (3.2) | 39 (2.8) | 0.2 | 0.18 − 0.34 | <0.001 |
|  | Mtwapa | 3971 (4.8) | 19 (1.4) | 0.1 | 0.04 − 0.11 | <0.001 |
|  | Sokoke | 2618 (3.2) | 11 (0.8) | 0.1 | 0.03 − 0.11 | <0.001 |
|  | Jaribuni | 1384 (1.7) | 12 (0.9) | 0.1 | 0.07 − 0.22 | <0.001 |
|  | Gede | 1996 (2.4) | 10 (0.7) | 0.1 | 0.04 − 0.13 | <0.001 |
| Survey Round | 1 | - | 869 (63.2) | 1 |  |  |
|  | 2 | - | 340 (24.7) | 0.4 | 0.39 − 0.49 | <0.001 |
|  | 3 | - | 167 (12.1) | 0.2 | 0.19 − 0.26 | <0.001 |

EVI; Enhanced Vegetation Index, LST; Land Surface Temperature, NTL; Night time Light, LULC; Land use land cover, OR: odds ratio; AOR: adjusted odds ratio; CI: confidence interval.

* median and Inter-Quartile ranges (IQR), units of these variables are ten times larger than presented. Actual median values are 50mm for rainfall, 307 K for LST, 64 for soil pH, 15g/kg for organic carbon content 620 mg/kg for aluminum content, and 130 mg/kg for iron content l, as well as IQR

### Figure S3: Pseudo R^2^ of each variable in the final model (model 2).

**
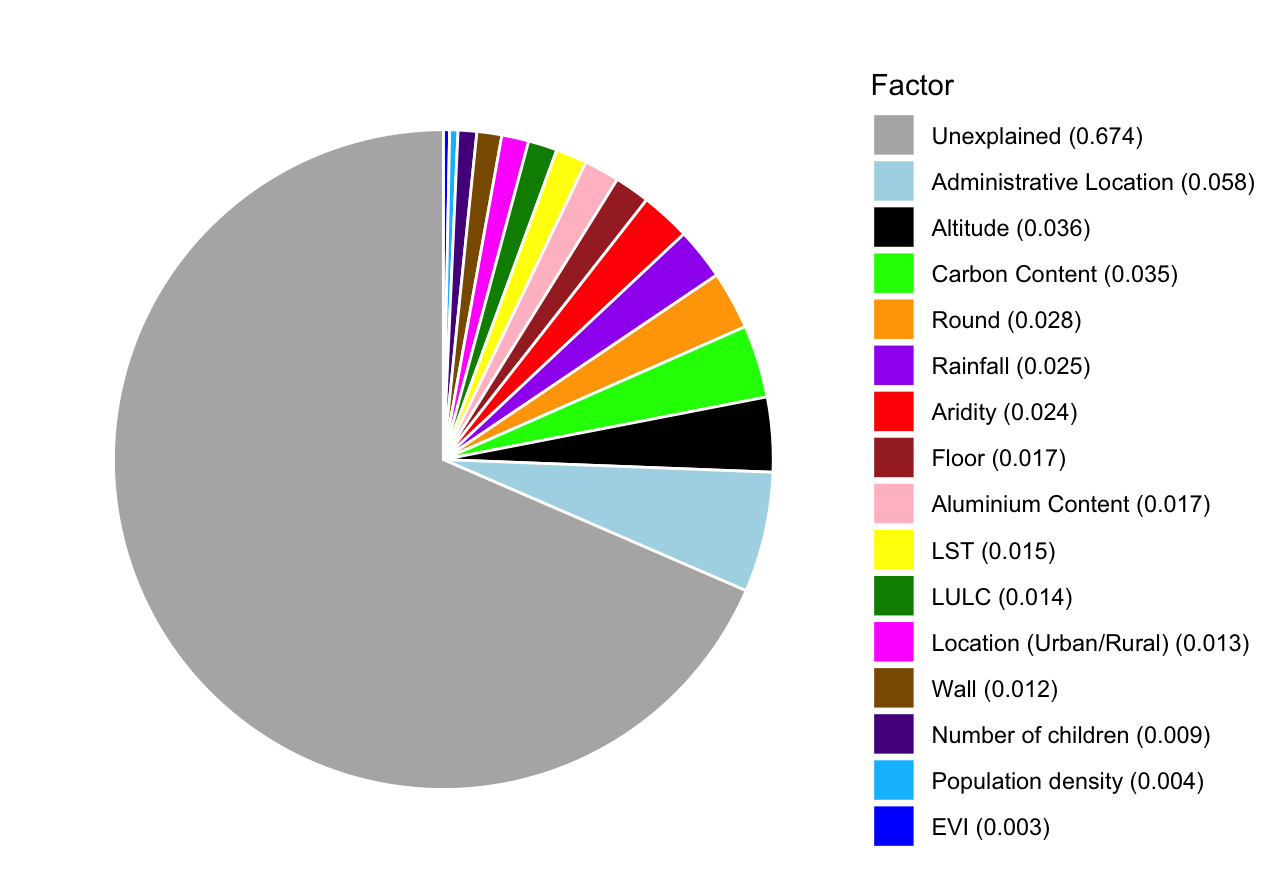
**
