# Supplementary material for: Prevalence, spatial and temporal distribution of tungiasis in the Kilifi Health and Demographic Surveillance System (KHDSS) in Kenya
Source: BMJ Glob Health. 2026 Mar 4;11(3):e020057. doi: 10.1136/bmjgh-2025-020057 (PMC12970124; doi:10.1136/bmjgh-2025-020057)
Supplement: online supplemental file 2 [file bmjgh-11-3-s002.docx]

### BMJ Global Health Author Reflexivity Statement

Adapted from Morton, B., Vercueil, A., Masekela, R., Heinz, E., Reimer, L., Saleh, S., Kalinga, C., Seekles, M., Biccard, B., Chakaya, J., Abimbola, S., Obasi, A. and Oriyo, N. (2022), Consensus statement on measures to promote equitable authorship in the publication of research from international partnerships. Anaesthesia, 77: 264-276. <https://doi.org/10.1111/anae.15597>

| **Study conceptualisation** | |
| --- | --- |
| 1. How does this study address local research and policy priorities? | Tungiasis is a disease of public health concern in Kenya where this study was conducted. The national policy and guidelines for control of tungiasis urges further research to understand the disease burden and distribution. |
| 1. How were local researchers involved in study design? | All of the authors, both Kenyans and non-Kenyans were involved in the study design from the proposal writing stage, through implementation, analysis and manuscript writing. |
| **Research management** | |
| 1. How has funding been used to support the local research team(s)? | The demographic surveillance research team is supported by the Wellcome Trust core funding grant. This study did not require any extra funding. |
| **Data acquisition and analysis** | |
| 1. How are research staff who conducted data collection acknowledged? | Some of the data was collected as part of an on-going demographic surveillance system run by KWTRP for the past 20 plus years. The field workers who collected the field data are acknowledged in the “acknowledgements” section. Spatial data was gathered from online repositories by the lead author, NO. |
| 1. How have members of the research partnership been provided with access to study data? | The combined dataset in excel was generated by NO, a Kenyan supported by his supervisors, AN and DW, both Kenyans which was then shared with LE, the British senior author and PI. |
| 1. How were data used to develop analytical skills within the partnership? | The study provided NO, a Kenyan master’s student and the lead author, with the experience of extracting data from the various systems, conducting the analyses and manuscript writing. SM, a Kenyan graduate student, gained experience with using these data to conduct the mapping. |
| **Data interpretation** | |
| 1. How have research partners collaborated in interpreting study data? | Regular online meetings were held to discuss and guide the analyses and then to interpret the results. |
| **Drafting and revising for intellectual content** | |
| 1. How were research partners supported to develop writing skills? | NO was mentored by AN to write the first drafts which were reviewed and edited by AN and LE. |
| 1. How will research products be shared to address local needs? | The findings have been shared back to the participating communities through their community liaison committees. A research dissemination meeting was held with all medical officers and public health officers responsible for the areas covered by the study as well as the County Health Director. |
| **Authorship** | |
| 1. How is the leadership, contribution and ownership of this work by LMIC researchers recognised within the authorship? | Seven of the eleven authors are from an LMIC including the lead author. |
| 1. How have early career researchers across the partnership been included within the authorship team? | The lead and second authors are Kenyan students. |
| 1. How has gender balance been addressed within the authorship? | Three of the authors are female. |
| **Training** | |
| 1. How has the project contributed to training of LMIC researchers? | The study provided NO, a Kenyan master’s student and the lead author, with the experience of extracting data from the various systems, conducting the analyses and manuscript writing. SM, a Kenyan graduate student, gained experience with using these data to conduct the mapping. |
| **Infrastructure** | |
| 1. How has the project contributed to improvements in local infrastructure? | This specific study has not improved local infrastructure, it has benefitted from the existing demographic surveillance platform established by Wellcome Trust. |
| **Governance** | |
| 1. What safeguarding procedures were used to protect local study participants and researchers? | The field workers and other members of the research team all receive comprehensive training and regular refreshers on research ethics and safeguarding, The institute (KWTRP) has a safeguarding policy which all field workers read and sign. The study was approved by the KEMRI scientific and ethics committee as well as the Oxford research ethics committee. |
